# Tribology of Coated 316L SS by Various Nanoparticles

**DOI:** 10.1155/2023/6676473

**Published:** 2023-08-22

**Authors:** Dhiaa J. Aldabagh, Thair L. Alzubadi, Akram F. Alhuwaizi

**Affiliations:** ^1^Department of Orthodontics, College of Dentistry, University of Baghdad, Baghdad 00964, Iraq; ^2^Department of Prosthodontics Dental Techniques, Al-Esraa University College, Baghdad 00964, Iraq

## Abstract

**Background:**

Nanocoating of biomedical materials may be considered the most essential developing field recently, primarily directed at improving their tribological behaviors that enhance their performance and durability. In orthodontics, as in many medical fields, friction reduction (by nanocoatings) among different orthodontic components is considered a substantial milestone in the development of biomedical technology that reduces orthodontic treatment time. The objective of the current research was to explore the tribological behavior, namely, friction of nanocoated thin layer by tantalum (Ta), niobium (Nb), and vanadium (V) manufactured using plasma sputtering at 1, 2, and 3 hours on substrates made of 316L stainless steel (SS), which is thought to be one of the most popular alloys for stainless steel orthodontic archwires. The friction of coated 316L SS archwires coated with Ta, Nb, and V plasma sputtering is hardly mentioned in the literature as of yet.

**Results:**

An oscillating pin-on-plate tribological test using a computerized tribometer was performed by applying a load of 1 N for 20 minutes under the dry condition at room temperature (25°C) to understand their role in the tribological behavior of the bulk material. Ta and Nb were found to reduce the friction of their SS substrate significantly (45 and 55%, respectively), while V was found to deteriorate the friction of its substrate. Moreover, sputtering time had no substantial role in the friction reduction of coatings.

**Conclusions:**

Nanocoating of 316L SS bulk material by Nb and Ta with a 1-hour plasma sputtering time can enhance dramatically its tribological behavior. Higher coating hardness, smaller nanoparticle size, intermediate surface coating roughness, and lower surface binding energy of the coatings may play a vital role in friction reduction of the coated 316L SS corresponding to SS orthodontic archwires, predicting to enhance orthodontic treatment.

## 1. Introduction

Developing biomaterials with less friction and high wear resistance is considered a substantial issue in the fabrication of long-standing and better-performance biomaterials in many medical fields, namely, orthodontics, focused on the reduction of treatment time and overcoming high orthodontic force abuse that may lead to unwanted treatment sequelae such as roots and alveolar bone resorption. Tribology, which comprises friction, wear, and lubrication, has an enormous impact on society as it may save 23% of the energy utilized [1]. Studying the tribology of biomedical materials is substantial to increase their durability and enhance their performance [2]. Materials no greater than 100 nm in size are usually contained in the nanotechnology field. The industrial, commercial, and agricultural sectors as well as the pharmaceutical and medical sciences have advanced technology, and this technology performed so swiftly [3, 4]. Nevertheless, compared to their bulk material, their properties at the nanoscale are vastly improved [5]. The possible risk associated with employing inorganic biomaterials can also be effectively reduced through surface modification [6]. Coatings are one of the most valuable protective materials for a variety of industrial uses [7]. Therefore, using hard coatings will result in the best thin coating contribution in tribology. Thermal spraying, chemical vapor depositions (CVD), physical vapor depositions (PVD), and ion beam-assisted deposition are important techniques for producing thin layer coating [8]. The optimization and improvement of coating hardness have a significant impact on other key features, including friction behavior and wear resistance [9].

A solid material's hardness can be determined as a gauge of how resistant it is to permanently changing shape when a constant compressive force is applied. Distortion can result from a variety of causes, including indentation, scratching, cutting, mechanical wear, or bending [10]. The hardness of a substance can be determined using a wide range of techniques. Since the creation of the first scientific hardness test by Austrian mineralogist Friedrich Mohs in 1812, mineralogists and geologists frequently use the Mohs scale [11]. The most popular technique for assessing a material's hardness is (quasi-)static indentation, which entails creating a persistent (plastic) indentation on the material's surface [12]. The test that was used to measure indentation hardness often determines its value. International standard methods have been developed for various techniques at the macro-, micro-, and nanoscale to deal with equivalent measured results [13]. Vickers and Knoop tests are the two main microhardness scale tests. These tests for indentation hardness gauge a material's resistance to being penetrated by a pyramid-shaped diamond indenter, and the hardness of a material corresponds with the depth to which an indenter will penetrate it under a given load and time [10].

Friction (FR) is the force that opposes motion when an object moves tangentially against another [14]. Like the modulus of elasticity, the coefficient of friction is not a property that is inherent in a material [15]. To comprehend the tribological properties of created biomaterials, it is essential to explain the wear and friction of those materials using the suitable test methodology. The most often used tests in the literature to look at the tribological behavior of metallic biomaterials are block-on-disc, ball-on-disc, and pin-on-disc [16]. The typical setting for these testing is either ambient [17] or dry sliding [18].

One of the main challenges to tooth alignment or retraction during orthodontic treatment is friction. Applying greater forces is one way to overcome it, although doing so could result in unfavorable anchoring loss. Altering the bracket design and archwire criteria and coating the wire surfaces with various biomaterials are further options that may help overcome slide resistance [19]. Depending on the type of ligation used to fasten orthodontic archwires to the orthodontic brackets, friction during clinical tooth movement will vary [20], as well as the substance of the orthodontic archwires and brackets [21]. Understanding and reducing friction when using orthodontic appliances has drawn more and more attention. Together with frictional forces, which may also result from the restriction of the tying mechanism, active tooth-moving forces can be used. Knowing friction may assist us in selecting a new appliance or improving the performance of an already installed appliance system [15]. One of the fundamental objectives of tribological research is to identify materials and material combinations with low coefficients of friction (CoF), low rates of wear, and extended lifetimes. Numerous strategies involving surface changes and coating systems have been explored to enhance the tribological performance of a given material [22]. The tribological performance of 316L SS substrates coated with Ta, Nb, and V by plasma sputtering has never been studied, as far as the authors are aware. Since 316L SS substrates are the most commonly used biomedical alloys in the production of orthodontic archwires, the goal of this research article is to characterize their tribological behavior, specifically their friction, after being nanocoated with Ta, Nb, and V, using plasma sputtering at various times (1, 2, and 3 hours).

## 2. Materials and Methods

Substrates of 316 L SS (15 × 15 mm and 2 mm thickness) were prepared under ASTM E3-95, after a sequence of abrasion cycles using 80–1000 grits paper, to get a scratch-free surface followed by polishing using a diamond suspension of 1–15 *μ*m to get a smooth mirrored surface. The nanoplasma sputtering was used for the coating process, which was carried out in a DC gas discharge with a 6 × 10^−2^ mbar low pressure. The DC power supply's voltage and discharge current were raised until plasma was produced at a 25 watt energy level (7 mA × 3.6 Kv). Targets for the production of nanoparticles were chosen to be sheets of highly pure niobium (Nb), tantalum (Ta), and vanadium (V). Surface characterization (structural, elemental, and topographical) of the deposited nanothin film layer was accomplished in our previous work [23], using X-ray diffraction (XRD), field emission scanning microscope (FESEM) combined with energy dispersive X-ray spectroscopy (EDS) detector, and atomic force microscopy (AFM). However, additional characterization was needed in the present study regarding the nanoparticle size of the performed coatings based on the assumption of its substantial role in the tribological behaviors of coating materials, and triple measurements of nanoparticle particle size for each analyzed specimen were registered using the highest magnification of (FESEM), of MIRA 3 XMU (TESCAN, Brno, Czech Republic) under low vacuum conditions, with an accelerating voltage of 15.0 KV, the view fields range from 1.38–8.30 *µ*m, and then averages were taken, in accordance to the methodology of Kumar et al. [24] and Farivar et al. [25].

### 2.1. Microhardness Vickers Test

According to ASTM E92 and more precisely E384, the Vickers microhardness test can be used in the assessment of thin films hardness [10]. Therefore, surface microhardness of coatings (Ta, Nb, and V at different sputtering times) and untreated 316L SS substrate was measured in the Material Engineering Department at the Technical University, Iraq, Baghdad, using HVS-1000 digital microhardness tester (Laryee Technology Quality Company, China) with an automatic loading and unloading cycle, according to ASTM E384 and ISO 6507. The applied load selected to be 100 gm (=0.98 N), which produces well-defined pyramidal indentation that can be assessed accurately among different tested specimens, at the same time, to simulate the in-service load during orthodontic tooth movement [26] supported by previous experimental conditions which were routinely used for the evaluation of the hardness of orthodontic components [27–29]. Four Vickers microindentations were registered at different sites for each tested specimen according to ASTM E384 standards (to prevent interaction between the hardened regions and the effects of the edge, the minimum distance between indentations should be at least 2.5 times the indentation diameter and away from the edge of the plate by the same amount). The collected data were arranged in tables and multiplied by 0.009807 to be converted to GPa which is the universal hardness unit used by SI units (International System of Units).

### 2.2. Friction Test

**Figure 1:**
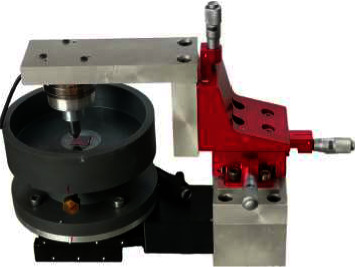
Modified tribometer system designed by Swaminathan and Gilbert [31].

The suitable test methodology is a critical point in tribological behavior of developed biomaterials. An ideal tool for measuring friction and/or wear in tribology is a pin-on-plate mechanical sliding tester [30], and methodology was performed according to ASTM G99, using a modified tribometer system designed by Swaminathan and Gilbert [31] with AITD analyzing software representing pin-on-plate wear and friction test (Figure 1) to measure CoF (*μ*), conducted under dry conditions at room temperature 25°C [16, 18, 32]. The process involves creating friction tracks on hard plates (coated 316L substrate by Ta, Nb, and V) to measure normal and tangential forces using special pin.

**Figure 2:**
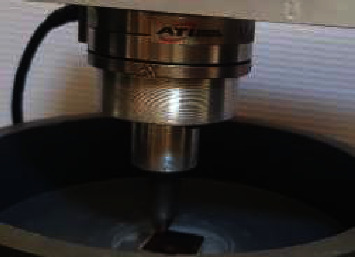
ATI-industrial automation loading cell.

The tribometer is composed of the following:

(1) Three-axis manual translation stage: HTIMS 301 (Technic Assembly Company, China), with a minimum scale reading of 0.005 mm and sensitivity of 0.002 mm, by which we can adjust carefully the position of the tested specimen and the amount of applied normal load on it as follows:
  - Vertical *Z* direction: adjust the amount of load applied by the pin on the tested specimen, usually attached to a 2-D load cell that holds the pin and measures both normal and frictional forces
  - Horizontal *X* direction: adjust the position of starting point of the friction track on the tested plate (nanocoated 316LSS)
  - Horizontal *Y* direction: adjust the distance between multiple friction tracks to keep them paralleled in *X*-horizontal direction
(2) Zaber motion control stage: type *x*-axis T-Is 13 m of 29 *µ*m accuracy and 0.0099 mm/s speed resolution (Zaber Technologies, Vancouver, Canada). A computer-controlled positioner makes automating submicron positioning quick, easy, and affordable, usually controlled by Zaber Console software, by which we can adjust the length of the friction track in addition to the speed and time of the motion during each oscillating friction cycle (Zaber script), hence determines the collected distance of the whole cycle.
(3) Loading cell: multiaxis force/torque sensor, type-Mini45 F/T transducer six-axis load cell, ATI Measurement Inc., USA, which measures all six components of force and torque. The maximum force measured by the loading cell is 16 N with a sensitivity of 0.001 N (Figure 2). It incorporates hardware temperature compensation capabilities that optimize the transducer's accuracy across a range of about ±25°C over ambient temperature and stable its sensitivity over temperature.
(4) Pin: a cylinder of 316L SS, 8 mm in diameter ends with a sphero-conical shape [33]. Before the settling of each friction cycle, the following should be monitored:
  - Firm mounting of the pin to the loading cell apparatus that is attached to the manually adjusted Z-direction part of the tribometer
  - Firm securing of the tested plate (specimen) to the sliding stage using a specially designed and prepared plastic holder using computer numerical control (CNC) technology
  - Cleaning of pin and plate with acetone and dry piece of cloth
  - Adjustment of Zaber console-specific script to apply specific criteria for each performed oscillating wear cycle; the following parameters were selected: sliding setup of 3 mm distance (represents the average width of orthodontic brackets) and 100 sliding oscillating cycles with a total distance of 600 mm for 20 min at 0.5 mm/sec. of speed, comparable to the nanotribological test performed by Grieseler [7]

The normal load applied on the nanocoated specimen was around 1 N to be comparable to in-service load equivalent to the optimum orthodontic force applied by the orthodontic appliance to move a tooth normally [26]. A tribological frictional test was performed for 30 specimens as follows: 3 specimens for untreated 316L substrates and 27 specimens (3 × 9) for Nb, Ta, and V coatings, subdivided equally into 3 subgroups corresponding to different sputtering times (1, 2, and 3 hours), and 5 friction cycles for each specimen were settled and then averaged to reduce methodology variations and increase the validity of the results; hence, a total of 150 friction cycles (tracks) were performed and analyzed in the present study. CoF was calculated using Fr/Fn, where Fr represents the frictional (tangential) force and Fn is the normal force.

Data collected were dealt with in two forms:

1. Average CoF of each subgroup and 316L SS along 20 min. of the friction cycle
2. Average CoF of each subgroup and 316L SS every 0.5 min. along the friction cycle to understand precisely the tribological behavior of each tested sample

## 3. Results

### 3.1. Particle Size

Table 1 presents the average nanoparticle size of coatings, and it is obvious that all of the measured particles were within the range of the nanoscale; however, the average V nanoparticles were found to be the highest (31.68 nm), while nanoparticles of Nb and Ta were comparable in size (19.71 nm and 18.73 nm, respectively).

### 3.2. Surface Microhardness

**Figure 3:**
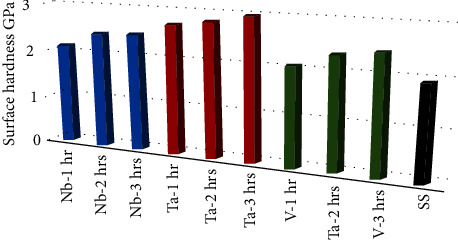
Surface microhardness of 316L SS and all nanocoatings sputtered at 1, 2, and 3 hours.

Figure 3 shows that the average surface microhardness of the 316L SS substrate was increased by Nb, Ta, and V coating at different coating times (1, 2, and 3 hours), with the Ta group having the highest average surface microhardness, followed by the Nb group, and the V group having the lowest average surface microhardness. Additionally, there was a trend of increasing surface hardness by increasing sputtering time, with the highest average surface hardness being in the 3-hour group and the least in the 1-hour group.

Table 2 presents the average surface microhardness of 316L SS in comparison to various used nanocoatings; generally, all of the coating groups (Ta, Nb, and V) and subgroups of different sputtering times (1, 2, and 3 hours) improved the surface microhardness of their SS substrates (1.91 GPa), and the highest was seen in Ta-3 hrs subgroup (2.96 GPa) while the least improvement was seen in V-1 hr (2.05 GPa). One-way ANOVA test presented significant differences among them statistically. Moreover, Tukey's post hoc test shows significant improvement of SS surface microhardness by the whole coating subgroup (regarding time difference) except for 1-hour Nb and V subgroups. Moreover, regarding material differences, Ta, Nb, and V coating improves significantly the surface microhardness of their SS substrates (Table 3).

### 3.3. Coefficient of Friction

**Figure 4:**
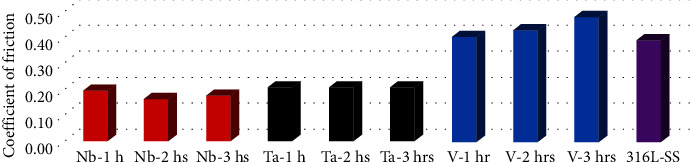
Mean coefficient of friction of various coating materials at different sputtering times and untreated 316L SS substrate.

Figure 4 shows an obvious reduction of coefficient of friction regarding Nb and Ta coatings at different sputtering times in comparison to their 316L SS substrate, while V coatings at different sputtering times showed a neglectable increase in coefficient of friction in comparison to their SS substrate.

Table 4 presents the comparison between the coefficient of friction of 316L SS substrate (0.38) and all nanocoatings (Ta, Nb, and V at 1, 2, and 3 hours sputtering time), and the highest was seen in the V-3 hrs coating (0.47) while the least was seen in the Nb-2 hrs coating (0.16). Moreover, regarding time difference, ANOVA statistical test showed significant differences between subgroups coated by Nb and Ta at different sputtering times and 316L SS, while V subgroups coated at the different sputtering times showed nonsignificant differences in comparison to their SS substrate; however, Tukey's post hoc tests (Table 5) showed nonsignificant statistical differences of frictional coefficient among each sputtered times (1, 2, and 3 hours) of the all coated subgroups revealing the ability to select 1-hr sputtered subgroup a representative of its group. Regarding coating materials difference—for each sputtering time—the ANOVA test showed significant differences between each coating (Nb, Ta, and V) at 1, 2, and 3 hours of sputtering times and untreated 316L SS substrates (Table 4); however, Tukey's post hoc tests in Table 5 showed nonsignificant differences of coefficient of friction between Nb and Ta at each sputtering times (1, 2, and 3 hours) indicating a comparable effect on the coefficient of friction after coating the SS substrates by either Nb or Ta coating materials. Moreover, a nonsignificant difference was found between the coefficient of friction between V coatings and their SS substrates at 1, 2, and 3 hours of plasma sputtering time, revealing the inability of V coatings to enhance the tribological behavior of their SS substrates which considered the most substantial goal of this research study.

**Figure 5:**
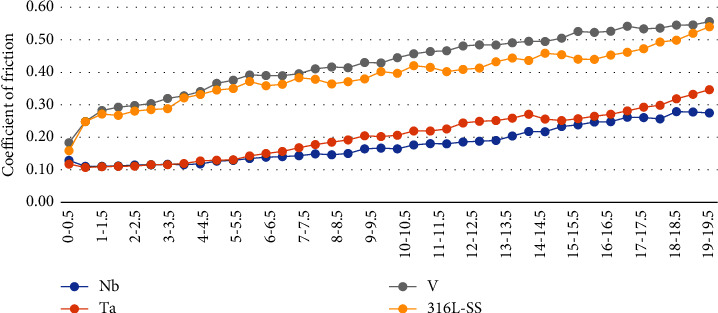
The average coefficient of friction of 316L SS substrate in comparison to the Nb, Ta, and V coatings (every 0.5 min along 20 min friction cycle).

Therefore, Figure 5 shows clearly an obvious reduction of coefficient of friction regarding Nb and Ta coatings in comparison to their SS substrate. Moreover, V coating showed a neglectable increase (deterioration) of coefficient of friction in comparison to their SS substrate (averages were taken every 30 sec. of friction cycles of each coating).

### 3.4. Correlation between Surface Microhardness and Coefficient of Friction

Table 6 presents a weak nonsignificant correlation between surface microhardness and coefficient of friction of Nb, Ta, and V coatings.

## 4. Discussions

A bulk material can be coated using a variety of surface treatment techniques; however, PVD, specifically sputtering, may be favored over others for the following reasons:

1. The process is physical, straightforward, and flexible, so the substrates' structural characteristics will not be impacted [34]
2. Delamination is reduced, and their substrates' adherence is improved [35, 36]
3. Proper thin film coating layers can be fabricated using broad spectrum metals and/or metal alloys [36, 37]
4. The sputtering time and/or circumstances can be adjusted to change the characteristics of coatings [37, 38]

**Figure 6:**
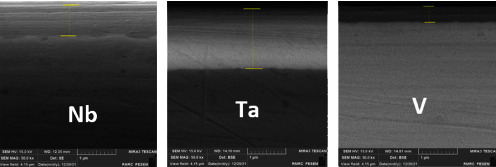
Cross section of Nb, Ta, and V nanocoating thin layers.

Hence, a thin, uniform layer of well-defined nanocoating was applied to the whole surface of the substrate; the thickness ranged from 197 nm to 1545 nm, with Ta coating and Nb coating having the thickest layers and V coating having the thinnest layers (Figure 6).

**Figure 7:**
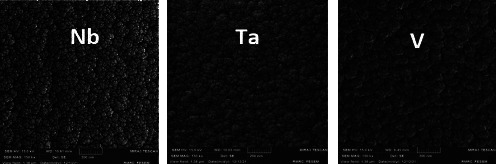
Topography of Nb, Ta, and V nanocoated particles.

Moreover, Ta, Nb, and V coating materials' sputtered nanoparticles were evenly and densely deposited over their substrates (Figure 7).

The effectiveness and longevity of produced biomedical materials are directly impacted by friction. Because of their high cost or improper tribomechanical characteristics, the usage of bulk materials is occasionally inappropriate, and surface coating may consider one of the best mechanisms for reducing friction, by applying coating material on cheaper substrates to enhance their performance and quality, which briefly stated that this is the main objective of this research. Moreover, there is some similarity between the hardness test and friction, since both of them encounter the moving of one surface against the other surface resulting in elastic deformation followed by plastic deformation on one or both of the moved surfaces. Hence, the hardness and friction of nanocoatings in the current study and 316L stainless steel substrates—the alloy most frequently used to make orthodontic SS archwires and brackets—were studied to evaluate the tribological behavior of these materials. According to the authors' knowledge, there was no study in the literature that dealt with the coating of 316L SS by Ta, Nb, and V using plasma sputtering method of the same experimental conditions. As a result, it was challenging to compare the present findings with other literature.

Starting with nanocoatings hardness, Grabco et al. [39] addressed that material hardness is a versatile physical characteristic that depends on a variety of internal and external conditions. Interaction between these components mostly accompanies the change in hardness scales (macro-, micro-, and nanoindentation measurement). In order to deal with comparable measured values, international standard criteria have been established for various methodologies at the macro-, micro-, and nanoscale. Indentation hardness magnitude also depends on the choice of an appropriate test employed to quantify it [13].

**Figure 8:**
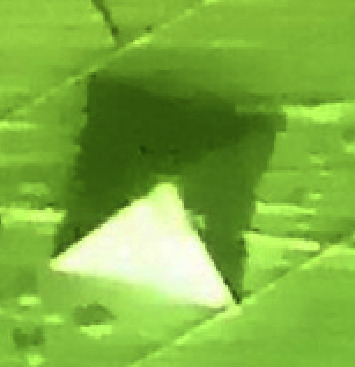
Micro-Vickers indentation of 100 mg load.

Four substantial factors may affect the accuracy of microhardness findings:

1. Types of indenter used: According to ASTM E92 and more precisely E384, Vickers and Knoop microhardness tests can be applied successfully for the thin film hardness assessment [10]. Although the Knoop indent is shallower (depth is approximately 1/30 diagonal length) than the Vickers indent (depth is approximately 1/7 the average diagonal length), making it more preferable for thin coatings assessment, contrariwise, the Knoop hardness varies with the load applied, and results are more difficult to convert to other hardness test scales than Vickers, in addition to that poorer visibility of the long diagonal tips on the Knoop indent than for Vickers indents, leading to under-sizing estimation [40]. Hence, Vickers indenter was selected in the present study to assess hardness variations of different coatings at different sputtering times in comparison to their 316L SS substrate hardness.
2. Applied load (hardness scale): Since thin film's hardness depends on the penetration depth, it is a vital approach to use a low load, hence reducing the influence of the substrate [7]. Moreover, Wasa et al. [41] reported that the microhardness scale is usually used to assess nanothin films hardness, while Broitman [10] found a hazy boundary between macro-, micro-, and nanoscale tribology experiments. Regarding the suitable load limit for microscale testing, there is some debate in the standards. According to ASTM E384 specification, the ideal load limit for microscale testing is 1–1000 gf (9.8 mN to 9.8 N) [42]. The ISO 14577-1 standard, on the other hand, states that loads less than 200 gf (1.96 N) are the most appropriate [43]. Hence, the Vickers microindentation test was selected in the present study using a 100 gm load, producing well-defined pyramidal indentation that can be assessed accurately among different tested specimens. Moreover, to simulate the optimum force usually used during orthodontic treatment that ranged from 50–100 gm [26], supported experimental conditions were routinely used in hardness assessment of orthodontic components in the literature [27–29, 44].
3. Thickness of the coat: The thin film's mechanical properties after it has been deposited on a substrate are usually influenced by the thickness of the coat and amount of indenter penetration (load applied); whenever penetration increased, more effect can be contributed to the substrate, therefore coating thickness considers a substantial factor in accurate hardness assessment. Bückle [45] was the first to propose the rule of thumb (film-thickness depth is usually indented by 10% corresponding to substrate thickness), which attempted to separate the contribution of the substrate from the total analyzed hardness at the microscale. Broitman [10] found that this rule is not applicable all the time, supported by others [46–48] who revealed that valid film hardness measurements for metallic thin films on rigid substrates may be achieved with indentation depths of under 20% of the film thickness. Moreover, Zak et al. [49] reported that the 10% film-thickness criterion is a relatively conservative guideline for hardness measurements while Chen and Bull [50] addressed that, in some situations, it is almost impossible to reliably determine coating hardness if the coating thickness is less than 100 nm due to the significant impact of the imperfect geometry and roughness of the indenter tip at minimal penetration, he overwhelmed, even if the indenter penetration is greater than 50% of film thickness. All coatings that were deposited in the present study ranged from 200 nm to 1600 nm thickness, revealing a reliable microhardness test was performed in the current study, expecting that there was no effect of substrate hardness on overlaying coating hardness and the analyzed surface microhardness belonged to the actual surface microhardness of coatings.
4. Visual perception of operator: More precisely in the case of low loads application, there will be a vital issue in defining where the indent tips are located. This requires proper illumination and frequent and patient adjustment of the optics for the best resolution, contrast, and careful focusing [51], as the low load usually gives a small blurred indentation size which is difficult to be assessed and is avoided in this study by doing a pilot study (trial applications of 10, 50, and 100 mg load to verify the appropriate load of perfect indenter tip identification); it is found that 100 mg gave a better well-defined indenter tip (Figure 8); moreover, all readings were performed by a same qualified operator.

The present study explored that the average surface microhardness of 316L SS substrate using the Vickers microhardness indentation test by applying a load of 100 mg was 1.91 ± 0.064 GPa. Moreover, performing this type of PVD nanocoating method led to significant improvement of the coating surface microhardness in varying degrees, being the best in Ta coating (35%), followed by Nb (22%), while V coating (18%) showed that the least improvement may be usually attributed to nanobiomedical materials' capacity to improve the tribological mechanical and behavior of their substrates [5], more precisely, depending on the hardness of elements in the periodic table.  Table 7 shows an obvious improvement in the surface microhardness of nanocoatings, compared with their relevant pure metal used as targets in the present study.

Depending on the findings addressed elsewhere by the same authors on comparable specimens [23], the elemental composition (wt. %) of the Ta nanocoating was the highest followed by the Nb coating, while the least was seen in the V coating group due to the variation in their surface binding energy and logically may interpret their comparable improvement of the surface microhardness of their 316L SS substrates, being the best in the Ta group followed by the Nb, while the least was seen in the V group, coincided with Tsongas et al. [9] who reported that coatings hardness is of crucial importance and is strongly associated with tribological behavior; meanwhile, its optimization and improvement may be attributed to the increased concentration of embedded nanoparticles, hence improving overall coating hardness. On the other hand, there was an obvious trend of increasing microhardness of all coatings with sputtering time elongation, being the highest for 3-hour sputtering time, decreasing with sputtering time reduction, which might be explained by a widely accepted physical phenomenon, and sputtering time has a directly proportional relation with the coating thickness, supported by our findings elsewhere [23]; therefore, the improvement of surface microhardness of all coatings with sputtering time prolongation was seen in the present study, which coincided with Zhang et al. [52], while Pena-Munoz et al. [53] found no relation between hardness and thickness when analyzing the effect of coating thickness on its hardness.

Regarding tribological behaviors of coatings, friction is a very complicated physical phenomenon that cannot be analyzed by a simple model. Almost every simple statement regarding frictional resistance can be combated with specific examples of the opposite meaning. Moreover, the materials' coefficient of friction is an attribute without dimensions and represents the amount of friction generated by two mating sliding materials that are determined experimentally.

It is crucial to underline that an oscillating, sliding setup was selected in the current study instead of a linear, unidirectional one to simulate the in-service condition during orthodontic tooth movement, and discontinuous and dynamic movement is expected rather than linear and continuous one [44]. As expected, the sputtering time of all coatings (Nb, Ta, and V) had a nonsignificant effect on their CoF supported by the findings of Li et al. [54], Hussein et al. [16], and Ma et al. [55] who found no clear linear relation between sputtering time and CoF since increasing it may lead to fluctuation of CoF up and down; therefore, the selection of the least performed sputtering time (1 hour) of all coatings in the present study could be more applicable from economic and resource-saving points of view. Meanwhile, the selection of the coating material type had a more substantial and crucial role on their tribological behaviors, and Nb and Ta coatings generally have a comparable effect on improving tribological features significantly, namely friction of their SS substrate being the best of the Nb group (55%) followed by Ta group (45%), while V coating group found to deteriorate the tribological feature, namely friction of its substrate, since it increased friction by 5%; although it was nonsignificant statistically, these manners of tribological behaviors might be attributed to the following interacting factors:

### 4.1. Particle Size

Commercial biomedical devices utilizing nanomaterials are currently commonplace, and their dimensional parameters play a significant role in determining their effect, property, and performance. Electron microscopy, still one of the main available analyzing techniques used to characterize and study nanoparticles [56], is considered the widely and preferable used metrological system in laboratories for characterizing the dimensional properties of nanoparticles due to its higher nanometric resolution that allows accurate measurement of nanometric scale structures; however, sample preparation procedures remain a keystone [57–59]. The present study showed that nanoparticle dimensions of Nb and Ta coatings were comparable and nearly half of V coatings, which may reflect their effect on the tribological behavior since both of them reduce the friction of their SS substrate nearly to half, while V coatings did not impact the coefficient of friction of their SS substrate positively, which may be attributed to the capability of smaller nanoparticles to form a tribofilm with stronger intrinsic mechanical properties or due to an increase in the ability of the nanoparticles passing through the contact, hence improving the tribological behavior of Nb and Ta coatings, coincided with Buranich et al. [60], Liu et al., [61], and Bhimaraj et al. [62], but in contrast to Zou et al. [63], who reported in their study that 30% of friction reduction may be gained by increasing nanoparticle size. Moreover, Rabaso et al. [64] found that nanoparticle size did not influence the coefficient of friction at all.

### 4.2. Surface Microhardness

Surface microhardness of the coated 316L SS substrates was improved in varying degrees in the present study according to the type of coating materials, being the best for Ta and Nb coatings and to a lesser extent by V coating; however, weak nonsignificant correlation was present between surface microhardness and coefficient of friction of all coatings (Nb, Ta, and v). In literature, there is a controversy associated with the effect of surface microhardness of coatings on their tribological properties, namely friction, and Buranich et al. [60] and Grieseler [7] found that harder surfaces usually improve the tribological behavior of coating, interpreted by the possible reduction of wear debris generated by harder surfaces, thus reducing third-body abrasion and friction between sliding surfaces, Moreover, Tsongas et al. [9] reported that there is a strong correlation between coating hardness improvement and the enhancement of tribological behavior of coatings which might be resulted from an increased concentration of embedded nanoparticles, while others found an inverse relation between hardness and frictional behavioral [65, 66]. On the other hand, Choy [15] reported that higher hardness is usually related to a low frictional coefficient; nevertheless, softer materials such as Teflon may have a low coefficient of friction.

### 4.3. Surface Roughness

Metal surfaces are genuinely rough, and asperities typically define how rough they are [67], and these asperities usually tolerate the total frictional load between mating surfaces and microscopically constituted the valuable interface region between two sliding solid surfaces which forms a very tiny fraction of the whole mating surfaces. According to a fundamental tenet of the physics of friction, flat, smooth surfaces may not be as smooth when analyzed microscopically or even may appear rough unexpectedly, and this constitutes the keystone of friction theory [68], and therefore, the amount of surface roughness is considered a substantial factor that affects the tribological behavior of coating [69], especially at low amounts of loads [70]. Choy [15] presented two main theories controlling friction, interlocking theory (explained by the stick-slip phenomenon, where the surfaces adhere together and then separate apart as a result of the presence of asperities seen at a microscopic level) and adhesion theory (seen at the ultramicroscopic level where adhesion of a sliding mating highly polished surfaces usually takes place). However, the obvious friction reduction effect is usually seen at the optimum surface roughness region [71]; however, this region does not refer to smoother surfaces at all; therefore, the old argument, smoother surfaces are usually associated with low friction, which is not applicable anyway nowadays; however, extremely rough or smooth one is usually associated with higher friction than intermediate one [15]; moreover, extreme smooth flat sliding surfaces may reach to a state of cold welding (adhering of two surfaces together) at which no movement of sliding mating surfaces may be expected; in our previous work, we found that all coatings (Nb, Ta, and V) reduced surface roughness of their 316L SS substrates in certain amounts, and the least amount of roughness was seen in the V group, expected to lay within the region of extreme smoothness, at which adhesive theory of friction is usually acting, leading to deterioration of its tribological behavior (increase frictional coefficient), while for Nb and Ta groups, moderate surface roughness was seen, hence laying within the optimal roughness region which predominates interlocking frictional theory (only discrete locations, where the asperities of one surface touch the other, cause meeting between tribe-pairs) leading to significant improvement of their tribological behavior via reduction of frictional coefficient nearly to the half, and a fact offered by Menezes and Kailas [72] provides a good explanation for the cause of these variations, who found that, for low values of the surface roughness, adhesion forces are usually dominated leading to increase CoF; however, at greater roughness values, interlocking or abrasion assumes a more significant role, improving CoF of their sliding mating surfaces, supported by Bera [73] who pointed out the importance of optimal region of surface roughness (intermediate zone) that enhances the tribological feature of coatings namely friction rather than extreme smoothness.

### 4.4. Surface Binding Energy

The surface binding energy of the nanocoatings is considered the most substantial factor controlling the surface composition of the coatings through conditioning the ion sputtering procedure, The surface binding energy (SBE) can be defined as the required applied energy necessary to remove an atom from the target surface layer during the sputtering technique in a vacuum, usually proportionate inversely to the sputtering yields of any target material [74]. Interestingly, it is found that SBE belongs to V metal has the highest value (512.3 eV), followed by Nb metal (202.4 eV), while the least value among the used targets was associated with Ta metal (21.8 eV) [75], expecting a logical variation in the elemental composition and thickness of the gained coatings being the lowest in the V group and the highest in Ta and Nb groups as found in our previous work [23], agreed with Arjunan et al. [76], Kudriavtsev et al. [74], and Dowling et al. [77] who pointed out the effect of the surface binding energy and its elemental composition (wt.%) in the coating after sputtering, and this may reflect the variations in the microstructure of the sputtered coatings (Ta, Nb, and V) which considered the most substantial factor affecting the tribological behavior of coating surface layer [78], being the best of Nb and Ta coatings in the present study, while the V coat did nothing or may deteriorate the tribological behavior of its 316L SS substrate.

## 5. Conclusions

Tribological behavior, namely, friction of nanocoated 316L SS substrates (the most popular used alloy for fabricating SS orthodontic wire and brackets) by Ta, Nb, and V, was analyzed using a computerized tribometer under the dry condition at room temperature, and the followings were concluded:

1. Nb, followed by Ta, found to improve significantly the tribological features, namely friction of their 316L SS substrates, while V was found to deteriorate it. Hence, either one of Nb or Ta coating can be used efficiently to reduce the frictional resistance of their 316L SS substrates that are used more commonly in the manufacturing of orthodontic appliances, improving its performance.
2. Plasma sputtering time has no role in improving tribological features of coated 316L SS substrates by Ta, Nb, and V; therefore, the least time (1 hour) could be the most appropriate one from the economic and resource-saving points of view.
3. Smaller nanoparticles size, higher surface microhardness, intermediate surface roughness (optimal roughness zone), and lower surface binding energy of the coatings may play a role in the enhancement of the tribological behavior (reducing the friction between SS orthodontic archwires and other components of orthodontic appliance especially brackets) after coating by Nb or Ta via plasma sputtering method, expecting to produce a more efficient orthodontic treatment in terms of time reduction and preventing unwanted sequelae of the orthodontic tooth movement as alveolar bone or/and dental roots resorption.
4. Surface microhardness of the 316L SS substrate was found to be improved significantly by Ta, Nb, and v coatings. However, weak nonsignificant correlation was found between surface microhardness and the coefficient of friction of all coatings.

## Figures and Tables

**Table 1:** The nanoparticle size of Ta, Nb, and V coatings sputtered with different times.

Coatings	Time	1st nm	2nd nm	3rd nm	Mean nm	SD±	Mean nm	SD±
Nb	1 hr	19.49	19.41	15.5	18.13	2.28	19.71	2.33
	2 hrs	19.16	16.32	20.39	18.62	2.09		
	3 hrs	18.56	23.5	25.1	22.39	3.41		

Ta	1 hr	23.71	22.29	21.7	22.57	1.03	18.73	3.97
	2 hrs	19.16	19.41	18.38	18.98	0.54		
	3 hrs	13.12	15.4	15.4	14.64	1.32		

V	1 hr	22	27.09	23.5	24.20	2.62	31.68	16.93
	2 hrs	17.1	18.81	23.43	19.78	3.27		
	3 hrs	50.98	42.54	59.67	51.06	8.57		

**Table 2:** Comparison of surface microhardness between 316L SS substrates and various coating materials at different sputtering times using one-way ANOVA.

Variables	1st reading	2nd reading	3rd reading	4th reading	Mean GPa	SD ±	ANOVA
Nb-1 hr	2.26	2.03	2.06	2.15	2.12	0.105	*F* = 14.783 *P* ≤ 0.001
Nb-2 hrs	2.23	2.45	2.54	2.46	2.42	0.134	
Nb-3 hrs	2.18	2.65	2.46	2.53	2.46	0.200	
SS	1.90	2.00	1.89	1.85	1.91	0.064	

Ta-1 hr	2.42	2.66	2.82	2.91	2.70	0.217	*F* = 29.580 *P* ≤ 0.001
Ta-2 hrs	2.57	3.03	2.81	2.82	2.81	0.190	
Ta-3 hrs	3.08	3.13	2.73	2.90	2.96	0.181	
SS	1.90	2.00	1.89	1.85	1.91	0.064	

V-1 hr	2.21	2.02	1.96	2.03	2.05	0.109	*F* = 37.977 *P* ≤ 0.001
V-2 hrs	2.24	2.30	2.34	2.38	2.32	0.061	
V-3 hrs	2.33	2.44	2.47	2.44	2.42	0.062	
SS	1.90	2.00	1.89	1.85	1.91	0.064	

Nb	2.22	2.38	2.35	2.38	2.33	0.075	*F* = 103.499 *P* ≤ 0.001
Ta	2.69	2.94	2.79	2.88	2.82	0.110	
V	2.26	2.25	2.26	2.28	2.26	0.014	
SS	1.90	2.00	1.89	1.85	1.91	0.064	

^*∗*^The mean difference is significant at the 0.05 level.

**Table 3:** Comparison of surface microhardness between 316L SS and various coating materials (Ta, Nb, and V) at different sputtering times using Tukey's post hoc tests.

Dependent variables	Independent variables		Mean difference	Sig.
Nb	SS	Nb-1 hr	−0.210	0.162
		Nb-2 hrs	−0.510^*∗*^	*P* ≤ 0.001
		Nb-3 hrs	−0.546^*∗*^	*P* ≤ 0.001

Ta	SS	Ta-1 hr	−0.793^*∗*^	*P* ≤ 0.001
		Ta-2 hrs	−0.898^*∗*^	*P* ≤ 0.001
		Ta-3 hrs	−1.050^*∗*^	*P* ≤ 0.001

V	SS	V-1 hr	−0.145	0.079
		V-2 hrs	−0.405^*∗*^	*P* ≤ 0.001
		V-3 hrs	−0.510^*∗*^	*P* ≤ 0.001

Coatings	SS	Nb	−0.423^*∗*^	*P* ≤ 0.001
		Ta	−0.916^*∗*^	*P* ≤ 0.001
		V	−0.353^*∗*^	*P* ≤ 0.001

^*∗*^The mean difference is significant at the 0.05 level.

**Table 4:** Comparison of the coefficient of friction between 316L SS and various coating materials (Ta, Nb, and v) at different sputtering times using one-way ANOVA test.

	1st specimen	2nd specimen	3rd specimen	Mean	SD ±	ANOVA
Nb-1 hr	0.16	0.21	0.21	0.19	0.027	*F* = 10.103 *P*=0.004
Nb-2 hrs	0.11	0.13	0.24	0.16	0.072	
Nb-3 hrs	0.18	0.16	0.16	0.17	0.012	
SS	0.44	0.38	0.33	0.38	0.053	

Ta-1 hr	0.22	0.20	0.20	0.21	0.009	*F* = 10.600 *P*=0.004
Ta-3 hrs	0.15	0.26	0.21	0.21	0.055	
Ta-3 hrs	0.25	0.22	0.15	0.21	0.048	
SS	0.44	0.38	0.33	0.38	0.053	

V-1 hr	0.29	0.40	0.50	0.40	0.083	*F* = 1.190 *P*=0.373
V-2 hrs	0.41	0.42	0.44	0.42	0.017	
V-3 hrs	0.45	0.52	0.45	0.47	0.041	
SS	0.44	0.38	0.33	0.38	0.053	

ANOVA	1 hr (Nb, Ta, V) and SS					*F* = 10.512 *P*=0.004
	2 hrs (Nb, Ta, V) and SS					*F* = 17.720 *P* ≤ 0.001
	3 hrs (Nb, Ta, V) and SS					*F* = 21.365 *P* ≤ 0.001

The mean difference is significant at the 0.05 level.

**Table 5:** Comparison of the coefficient of friction between 316L SS substrates and various coating materials (Ta, Nb, and V) at different sputtering times (1, 2, and 3 hours) using Tukey's post hoc tests.

Dependent variables	Independent variables		Mean difference	Sig.
Nb	SS	Nb1	0.190^*∗*^	0.017
		Nb2	0.223^*∗*^	0.007
		Nb3	0.223^*∗*^	0.007
	Nb1	Nb2	0.033	0.895
		Nb3	0.033	0.895
	Nb2	Nb3	0.000	1.000

Ta	SS	Ta1	0.177^*∗*^	0.008
		Ta2	0.177^*∗*^	0.008
		Ta3	0.177^*∗*^	0.008
	Ta1	Ta2	0.000	1.000
		Ta3	0.000	1.000
	Ta2	Ta3	0.000	1.000

V	SS	V1	−0.013	0.993
		V2	−0.040	0.863
		V3	−0.090	0.362
	V1	V2	−0.027	0.953
		V3	−0.077	0.486
	V2	V3	−0.050	0.770

1 hr	SS	Nb	0.223^*∗*^	0.012
		Ta	0.177^*∗*^	0.041
		V	−0.013	0.994
	Nb	Ta	−0.047	0.813
		V	−0.237^*∗*^	0.009
	Ta	V	−0.190^*∗*^	0.029

2 hrs	SS	Nb	0.190^*∗*^	0.006
		Ta	0.0177^*∗*^	0.009
		V	−0.040	0.752
	Nb	Ta	−0.013	0.986
		V	−0.230^*∗*^	0.002
	Ta	V	−0.217^*∗*^	0.003

3 hrs	SS	Nb	0.223^*∗*^	0.005
		Ta	0.0177^*∗*^	0.019
		V	−0.090	0.265
	Nb	Ta	−0.047	0.735
		V	−0.313^*∗*^	0.001
	Ta	V	−0.267^*∗*^	0.002

^*∗*^The mean difference is significant at the 0.05 level.

**Table 6:** Correlation between surface microhardness and coefficient of friction.

Variables		Surface microhardness		
CoF	Coatings	Nb	Ta	V
	Pearson correlation	0.09	−0.575	0.153
	Sig. (2-tailed)	0.817	0.105	0.694
	*N*	9	9	9

Correlation is significant at the 0.01 level (2-tailed).

**Table 7:** Comparison of the metal hardness between the pure Nb, Ta, and V metal targets and their corresponding nanocoatings.

Materials	Hardness of pure metals (GPa)	Average hardness of related coatings (GPa)
Nb	1.32	2.33 ± 0.08
Ta	0.87	2.82 ± 0.11
V	0.63	2.26 ± 0.10

## Data Availability

The data used to support the findings of this study are available from the corresponding author upon request.
